# Qualitative Interdisciplinary Learning Reviews of Non-COVID-19 Patients’ Journeys During the COVID-19 Pandemic

**DOI:** 10.7759/cureus.14378

**Published:** 2021-04-09

**Authors:** Matthew Mo Kin Kwok, Clinton Y Tsang, Lisa Stewart, Norm Greenway, Lisette Montessori

**Affiliations:** 1 Department of Emergency Medicine, Faculty of Medicine, University of British Columbia, Vancouver, CAN; 2 Department of Emergency Medicine, Richmond Hospital, Vancouver Coastal Health, Richmond, CAN; 3 Quality and Patient Safety, Vancouver Coastal Health, Richmond, CAN

**Keywords:** interdisciplinary learning review, quality assurance & improvement, multidisciplinary review system, covid-19 pandemic, patient journey, quality review, healthcare system, system impact, opportunities for improvement

## Abstract

In the early months of 2020, hospital processes were changed in response to the coronavirus disease 2019 (COVID-19) pandemic. We present one community hospital’s experience in conducting a series of interdisciplinary learning reviews (ILRs) on non-COVID-19 patient’s journeys during the early months of the pandemic. An ILR is a method of reviewing medical records using a system lens to identify system-level opportunities for improvement. Using the ILR method, we identified several opportunities for improvement in caring for our patients.

## Introduction

The coronavirus disease 2019 (COVID-19) pandemic has severely disrupted the lives of billions around the world. Out of the many profound impacts of the pandemic, healthcare systems have been among the most challenged to adapt in ways that not only meet the healthcare needs of the population they serve but also prevent the spread of COVID-19. While the long-term impacts of the pandemic and infection control measures put in place on population health have not yet been fully appraised, early research has provided evidence of these impacts.

One of the concerns during the pandemic was the delayed provision of appropriate care due to the need to first establish patients’ COVID-19 status, resulting in an increased wait-time for definitive medical or surgical treatments [1-5]. Delay of care was also associated with the public’s reluctance to seek medical care due to concerns about contracting COVID-19 in medical settings. Emergency departments in Canada and around the world experienced a decrease in the number of visits not related to COVID-19 [6-7]. Such a decrease was especially noted with certain clinical populations, for example, neurological and cardiac patients [8-9]. However, the proportion of these patients who needed to be hospitalized was found to be higher [10], and patients were found to present with more severe illnesses and medical complications [11], suggestive of delays in seeking medical care.

In addition to the delay in care, the pandemic also significantly changed how care was delivered. To minimize risks for disease spread, hospital visitation restrictions were implemented in many hospitals, ranging from limiting the number of visitors allowed, allowing essentials visits only, to outright visitor bans [12]. This gave rise to a reduction in psychosocial support by caregivers and family during patients’ journeys and resulted in not only compromising the quality of care and patient health outcomes [12-13] but also jeopardizing the psychological well-being of the family and the principles of family-centered care [14-15]. 

Rigorous use of personal protective equipment (PPE) was also introduced to protect healthcare workers and patients from potential exposure to COVID-19. However, with facial expressions being obscured by face masks and eye protection, communication with patients was significantly challenged, especially those with hearing and/or cognitive impairment [16-17]. Suboptimal communication with patients hindered the building of rapport with patients [18], impeded clinical assessments [19], and was associated with negative health outcomes [16].

In the summer of 2020, we took the opportunity to investigate and identify possible impacts and opportunities for improvement on our patients’ journeys due to the public health or system-level modifications that occurred due to the COVID-19 pandemic. The focus of this exercise was to have a system-level understanding of the impact of the pandemic response of our hospital in the early spring of 2020 when we had limited information in the first wave of the COVID-19 pandemic. The goal was to provide insight into the impacts of the decisions so that we could make improvements in patient safety and quality of care.

An interdisciplinary learning review (ILR), also known as a multidisciplinary review system (MRS), is a method to review charts using a system lens to identify system-level opportunities for improvement. It was developed by Huddleston J et al. at Mayo Clinic [20], where the goal for initiating this method of review was to create a meaningful mechanism to learn about system-level patient safety and quality of care that is understandable, measurable, and improvable.

## Materials and methods

The hospital setting is a university-affiliated community teaching hospital located in British Columbia (BC), Canada. The hospital receives approximately 60,000 per year of emergency department (ED) visits and delivers over 2000 newborns per year at our birth center. It is the nearest hospital to an international airport serving over 25 million passengers from over 100 countries annually [21]. As a result, it frequently encounters patients as returning travelers or visitors from other countries.

The ILR methodology was used to qualitatively examine patients with non-COVID-19-related medical illnesses requiring admission to the hospital in the midst of the first wave of the COVID-19 pandemic in BC.

**Figure 1:**
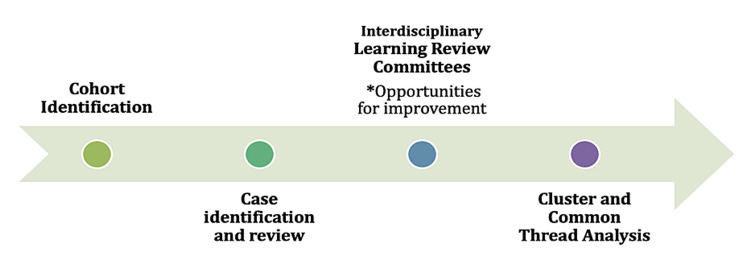
Interdisciplinary Learning Review Process

The ILR process involves four stages (Figure 1). They are 1) Cohort Identification; 2) Case Identification and Chart Review; 3) Interdisciplinary Learning Review Committee Meetings; and 4) Cluster/Common Thread Analysis.

1) Cohort identification

We selected seven patients’ journeys in April 2020, which were common non-COVID-19 cases at our hospital for review. They were:

Case 1: Palliative Care

Case 2: Stroke

Case 3: Suicidal Ideation

Case 4: Gastrointestinal Bleed

Case 5: Hip Fracture

Case 6: Spontaneous Vaginal Delivery

Case 7: Gastrointestinal Surgery

2) Case identification and chart review

Each chart was reviewed by one physician and one or two nurses or allied health providers, who had clinical knowledge in the patients’ journey examined but did not provide direct care to the patient. Standardized training, which included online and in-person sessions, was provided to 18 reviewers, including seven physicians, nine nurses, and two allied health providers. The focus was to identify system-level opportunities for improvement in the specific patient journey being examined. All qualitative data were logged into a secured web-based platform.

3) Interdisciplinary learning review committee meetings

An ILR committee meeting followed the chart review for each of the seven patient journeys. The meetings were attended by operational leaders, directors, managers, physicians, nurses, pharmacists, allied healthcare workers, and other frontline care providers to engage in an interdisciplinary discussion about the opportunities for improvement (OFI) identified in the chart review. The reviewers acted not only as presenters but were engaged in active dialogue with other care providers and administrators of the healthcare team not involved with the chart review. The goal was to find consensus on the OFIs in each of the seven patient journeys. Two scribes documented the topics of discussion, what was said, and the tone of the discussions. A total of seven ILR committee meetings were completed for this cohort.

4) Cluster/common thread analysis

We were unable to fully complete a comprehensive cluster and common thread analysis of the patients’ journeys examined due to time constraints, resource allocations, and small sample size. However, after the completion of the ILR committee meetings, all OFIs were reviewed and categorized by a smaller committee of ILR leaders. Consensus was reached, and common OFI clusters and themes were identified. The OFI clusters and themes were prioritized, grouped, and presented to frontline care teams and operational leaders.

## Results

Eight common themes of opportunities for improvement from the early pandemic response were identified in our interdisciplinary learning review process.

1) Social distancing and isolation

Social distancing as a public health measure appeared to have an adverse impact on the mental health of the community. We identified cases of psychiatric illnesses greatly exacerbated by an increase in social isolation. Furthermore, social isolation was an issue in certain vulnerable segments of the population. Elderly patients were often isolated at home without social contacts and received less support from the community. Parents of newborn children received less family and social support compared to pre-COVID-19 time due to fear of disease spread. Social distancing precautions, although important in containing the spread of COVID-19 infection, contributed adversely to the mental health and social needs of the community.

2) Reluctance in seeking medical care

Due to fear of contracting COVID-19, individuals accessing services were either reluctant to seek medical care, presented to medical facilities in more advanced stages of their illness, or made riskier health care decisions. For example, we discovered cases of delayed presentation of stroke and sepsis to the emergency department. We found pregnant patients with a lengthy time of active labor being reluctant to present to the hospital or choosing to attempt a vaginal birth post-cesarean section at home. We uncovered that the hesitancy to seek medical care was due to concern about the risk of COVID-19 infection in the hospital setting.

3) Case finding and obstacles in care of non-COVID-19 illnesses

Clinicians often found it difficult to identify people with COVID-19. Patients with COVID-19 presented to the hospital with symptomatology often similar to other disease processes. Many patients required a COVID-19 swab to rule out the presence of COVID-19 infection. This was of particular importance for patients requiring potential aerosolized generating medical procedures. As a result, many patients encountered obstacles in receiving definitive care for their non-COVID-19 illnesses.

4) Visitation and barriers to family members

Limiting visitors appeared to have unintended consequences in patient care, experience, and journey. Although limiting visitors in the hospital was an important strategy in preventing the spread of COVID-19, we discovered that this policy created barriers between patients’ advocates and the healthcare team. Healthcare team members found it challenging to provide patient status updates to the next of kin or power of attorney. In addition, healthcare team members found that visitors had an important role in the healing process of patients affecting both physical and mental health. Limiting visitors had a negative impact on the patients’ hospital journey. This impacted patients’ rates of recovery and experience in the hospital.

5) Communication

Various factors, including more regular and robust use of personal protective equipment, resulted in barriers in communication between health care team members to patients and between health care team members to other health care team members. Masks created a barrier in verbal and non-verbal communication. It was more difficult to manage patients with low English proficiencies due to increased struggles in having appropriate interpreters. Virtual consultations made it challenging to send clinical notes to the hospital for transitioning of care. Communication became complicated because of COVID-19 pandemic measures.

6) Clinical assessment and staffing

Due to robust infection control precautions, providing frequent clinical assessments to patients became a challenge. New processes due to COVID-19 often resulted in increased staffing needs in the care of patients. Clinicians often experienced actual or perceived pressure in discharging patients and therefore focused on one medical problem while other medical issues were overlooked. Concerns for COVID-19 transmissions resulted in clinical assessment obstacles, pressure to discharge patients, and placed a strain on the health care team.

7) Closure or suspension of services

Many medical facilities and programs were suspended, especially in the early stages of the COVID-19 pandemic. Group therapies were canceled, and elective procedures were postponed. Certain public health programs were suspended due to changes in resource allocation to support the COVID-19 pandemic response. These interruptions, at times, resulted in a worsening of clinical status and hospital admissions. The closure or suspension of medical services had a negative impact on patients’ illnesses and their medical care.

8) Follow-up and transition of care

Follow-up and obtaining resources for a safe discharge from an acute care setting was difficult during the pandemic. This was, in part, due to the suspension of medical and community support services. Many family physician offices were closed and access to primary care physicians became a challenge. Also, patients’ family members struggled in obtaining health care resources such as medical equipment or medications. There were also obstacles in returning patients to long-term care facilities, as resources in these facilities were often strained. Situations mentioned often resulted in prolonging patients’ length of stay in the hospital and delaying patients' return to the community.

## Discussion

In our ILRs of non-COVID-19 patients’ journeys during the COVID-19 pandemic, we identified eight areas of opportunities for improvement. These opportunities for improvement were identified and categorized by 18 trained ILR reviewers and involved seven sessions of review committee meeting forums consisting of physicians, nurses, allied health care clinicians, operational managers, and directors. The ILR process we experienced was collaborative, and we had constructive learnings to improve patient care in our clinical setting.

The opportunities for improvement identified in the ILR process were consistent with the challenges faced by healthcare workers and systems around the world in our literature review. For example, the need to first establish patients’ COVID-19 status and patients’ reluctance to seek medical care due to concerns for COVID-19 infection delayed and interrupted the usual flow of care [1-3,10-11]. Social distancing and visitation limitations induced psychological burdens on patients and families and impacted the recovery process [12-13]. The rigorous application of PPE created barriers to the usual communication between the healthcare team and patients, especially those who have special communication needs [16-17].

The British Columbia Health Quality Matrix characterizes the quality of care by six attributes: respect, safety, accessibility, appropriateness, effectiveness, equity, and efficiency [22]. In these regards, the COVID-19 pandemic and the changes it brought to healthcare access and delivery had significantly challenged healthcare systems in British Columbia, including ours, to maintain the quality of care while containing the spread of the disease.

## Conclusions

We report our experience in conducting a series of ILRs on non-COVID-19 patients’ journeys during the early months of the pandemic. We identified several opportunities for improvement in caring for our patients through this chart review process. We anticipate that our learning from the review process would assist clinicians and organization leaders in better understanding some of the challenges and opportunities for improvement in our health care setting as we continue to care for our patients in the midst of the COVID-19 pandemic.
